# A Sea of Change

**DOI:** 10.1371/journal.pbio.1001683

**Published:** 2013-10-15

**Authors:** Jonathan Chase

**Affiliations:** Freelance Science Writer, Saint Louis, Missouri, United States of America

[Fig pbio-1001683-g001]When most people think about the influence of burning fossil fuels on the global ecosystem, they usually think of the “greenhouse effect” caused by a skyrocketing increase in the levels of CO_2_ in the atmosphere, which traps a higher percentage of radiant energy and raises global temperatures. Despite politically charged opposition to an overwhelming scientific consensus, public concern regarding global warming and associated climatic change has grown substantially in recent years. Solutions ranging from “cap and trade” economic policies, development and use of alternative energies, and minimizing destruction of forested ecosystems that absorb CO_2_ are now part of our everyday parlance.

**Figure pbio-1001683-g001:**
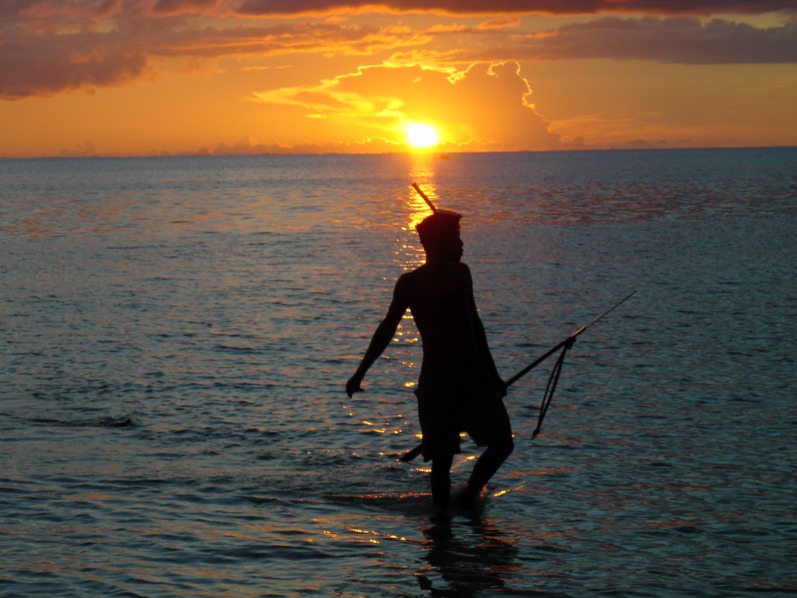
Mora and colleagues estimate that by the year 2100 rising carbon dioxide levels in the atmosphere will severely impact 470–870 million people who depend on the oceans for their livelihoods via adverse effects on oceanic biogeochemistry. *Image Credit: Joshua Cinner.*

Less frequently discussed, though no less important, are the many subsidiary effects of increased CO_2_ on the global ecosystem, including those that can dramatically influence humans. More than two-thirds of the planet is covered by ocean, and we humans extract a large proportion of our food and other resources from the sea. Awareness of the influence of increased atmospheric CO_2_ on oceanic resources is typically limited to concerns over loss of sea ice and rising sea levels from warming temperatures. However, the higher temperatures that result from increased atmospheric CO_2_ can also change oceanic circulation and stratification, as well as increase the rate of photosynthesis by algae, altering the productivity of the system upon which the entire food web is based. Furthermore, altered temperatures and productivity can influence the level of dissolved oxygen in the water, which is critical for the respiration of most organisms. Finally, perhaps the most important influence of increased CO_2_ in the ocean has more to do with chemistry than climate; increased levels of CO_2_ dissolved into seawater creates carbonic acid, ultimately reducing oceanic pH. In fact, the pH of the world's oceans has already decreased by 0.1 since the industrial revolution (representing a ∼25% decrease, since pH is measured on a logarithmic scale). This reduces the availability of calcium carbonate in seawater, upon which a wide variety of organisms such as corals and shellfish depend for skeletal material.

When the effects of manmade CO_2_ emissions on seawater pH, temperatures, productivity, and oxygen are examined in total, the magnitude of change in oceanic waters over the next 100 years is projected to exceed that observed at any time in the last 20 million years. What is less well-known, however, is just which parts of the world's oceans will be more vulnerable to these changes, which ecosystems and organisms will be exposed to these changes, and how those changes might impact human populations that depend on oceanic resources. In this issue of *PLOS Biology*, Mora and colleagues accomplish just this through an interdisciplinary collaboration between climate modelers, biogeochemists, ecologists, and social scientists.

After calibration, Mora and colleagues used the most recent and robust models of projected climate change (as part of the Coupled Model Intercomparison Project Phase 5 in the Fifth Assessment Report of the Intergovernmental Panel on Climate Change) to examine how oceanic biogeochemical parameters are likely to change by the year 2100. They projected change under two different CO_2_ emissions scenarios—“business as usual,” leading to an expected 900 parts per million (ppm) of CO_2_ in the atmosphere (more than double today's level and triple the level from pre-industrial times), and one of intense CO_2_ mitigation action, leading to only 550 ppm CO_2_ and more modest climate change.

Though there was variation in the projections for each emissions scenario depending on how the climate system was modeled, the overall trends from all model scenarios were similar. For both emissions scenarios, sea surfaces across the globe were projected to increase in temperature with concomitant declines in pH, dissolved oxygen, and phytoplankton production; the magnitudes of expected change predictably depended on the intensity of emissions control. In contrast to the sea surface, most biogeochemical parameters were projected to change considerably less on the sea bottom. In addition, changes in biogeochemical parameters were projected to differ between regions. For example, pH was projected to be least changed in tropical areas, whereas temperature and productivity were projected to change least in temperate areas. In all, the majority of the ocean system, both surface and floor, was projected to change one or more of these biogeochemical parameters towards values that tend to reduce ecosystem functions (e.g., reductions in productivity or pH).

Next, Mora and colleagues examined the co-occurrence of these biogeochemical changes among areas of the ocean representing different habitat types and hotspots of biodiversity within those habitat types. As might be expected, given the differences in exposure in different oceanic realms, deeper water habitats (e.g., sea mounts, deep sea benthic areas) were projected to experience less change than shallower water habitats like coral and rocky reefs, mangroves, and sea grass beds. Furthermore, hotspots of corals and mangroves were projected to be exposed to less biogeochemical change, while hotspots of whales, pinnipeds, squid, and krill were projected to experience much greater shifts in biogeochemistry.

As a final step, Mora and colleagues examined which groups of people living near the world's coasts would be exposed to the higher or lower degrees of projected biogeochemical change, the degree of dependency of these people on oceanic resources, and the potential for societal adaptation to environmental change (quantified by per capita gross domestic product, indicating the ability to access alternative resources). They projected that when assuming only moderate CO_2_ increases, ∼1.4 billion people will be exposed to medium-to-high oceanic change by 2100; of those, ∼690 million will be in countries with a medium-to-high ocean dependence and ∼470 million of these will also live in low-income countries. Under the “business as usual” scenario, however, ∼2 billion will live near oceans with medium-to-high biogeochemical change, of which 1.12 billion will live in countries with medium-to-high dependence on the ocean and ∼870 million will have little ability to adapt.

Of course, throughout, Mora and colleagues are careful to recognize that just because the biogeochemistry of the ocean changes, that does not mean those changes are necessarily “bad”; this is particular important when bringing humans into the equation. For example, it's difficult to predict just how changing temperature, productivity, oxygen, and/or pH will influence important subsistence fisheries upon which the less affluent and adaptable societies may depend. Indeed, in the game of global change, for every species that loses, others will gain. However, many of the changes projected by Mora and colleagues, such as reduced pH and productivity, are known to have negative consequences for the functioning of many important ecosystems that house high levels of biodiversity and supply a number of services to humans. While change can sometimes be good, humans, especially those from populations that rely heavily on oceanic resources for subsistence, are likely to be severely impacted by changing oceanic biogeochemistry within a relatively short time period. The good news is that this fate is still in our hands. However, it is clear that “business as usual” will wreak much greater havoc on the future of our oceans than will more moderate, but entirely doable, CO_2_ mitigation strategies.

**Mora C, Wei C-L, Rollo A, Amaro T, Baco AR, et al. (2013) Biotic and Human Vulnerability to Projected Changes in Ocean Biogeochemistry over the 21st Century.** doi:10.1371/journal.pbio.1001682

